# Isolation of a Methicillin-Resistant *Staphylococcus pseudintermedius* Strain from a Domestic Dog with Vulvovaginitis: A Potential Public Health Concern?

**DOI:** 10.3390/antibiotics15050480

**Published:** 2026-05-09

**Authors:** Lorenzo Pace, Valeria Rondinone, Laura Del Sambro, Viviana Manzulli, Stefano Castellana, Luigina Serrecchia, Angelica Bianco, Beatrice Maffei, Leonardo Marino, Antonio Petrella, Domenico Galante

**Affiliations:** Istituto Zooprofilattico Sperimentale della Puglia e della Basilicata, Via Manfredonia 20, 71121 Foggia, Italy; lorenzo.pace@izspb.it (L.P.); laura.delsambro@izspb.it (L.D.S.); viviana.manzulli@izspb.it (V.M.); stefano.castellana@izspb.it (S.C.); luigina.serrecchia@izspb.it (L.S.); angelica.bianco@izspb.it (A.B.); beatrice.maffei@izspb.it (B.M.); leonardo.marino@izspb.it (L.M.); antonio.petrella@izspb.it (A.P.); domenico.galante@izspb.it (D.G.)

**Keywords:** *Staphylococcus pseudintermedius*, One Health, multidrug resistance, WGS

## Abstract

**Background:** *Staphylococcus pseudintermedius* is a major opportunistic pathogen of dogs and the primary cause of canine pyoderma and other infections. The global emergence of methicillin-resistant *S. pseudintermedius* (MRSP) represents a significant challenge in veterinary medicine due to its frequent multidrug-resistant phenotype and limited therapeutic options. **Methods:** We describe the phenotypic and genomic characterization of an MRSP isolate recovered from a vaginal swab of an 11-year-old dog diagnosed with vulvovaginitis in southern Italy. Antimicrobial susceptibility testing was performed using broth microdilution according to CLSI VET01S guidelines. Whole-genome sequencing was conducted to determine sequence type and antimicrobial resistance determinants. **Results:** The isolate was identified as *S. pseudintermedius* by MALDI-TOF MS and confirmed by genomic analysis. Multilocus sequence typing assigned the strain to sequence type ST2333. Phenotypically, the isolate exhibited multidrug resistance, including resistance to β-lactams, macrolides, lincosamides, tetracyclines, aminoglycosides, fluoroquinolones, and trimethoprim–sulfamethoxazole, while remaining susceptible to amikacin, rifampicin, florfenicol, and vancomycin. Whole-genome sequencing confirmed the presence of mecA and additional resistance determinants consistent with the observed phenotype. **Conclusions:** This report suggests the possible occurrence of an MDR MRSP ST2333 lineage in southern Italy and highlights the importance of combined phenotypic and genomic surveillance to support antimicrobial stewardship in veterinary medicine within a One Health framework.

## 1. Introduction

*Staphylococcus pseudintermedius* is a Gram-positive, coagulase-positive bacterium belonging to the *Staphylococcus intermedius* group [1] and is widely recognized as one of the most prevalent commensal microorganisms colonizing the skin and mucous membranes of dogs and cats [2]. Under normal conditions, this bacterium exists as part of the resident microbiota without causing disease. However, when host defenses are compromised due to factors such as skin barrier disruption, underlying allergic conditions, hormonal imbalances, immunosuppression, or invasive medical procedures, *S. pseudintermedius* can act as an opportunistic pathogen [3].

Clinically, *S. pseudintermedius* is most frequently associated with canine pyoderma, otitis externa, wound and post-surgical infections, urinary tract infections, and reproductive tract disorders. In rare cases, it may also cause severe systemic infections, including bacteremia [4]. Over the past two decades, the increasing emergence of methicillin-resistant *S. pseudintermedius* (MRSP) has become a major concern in veterinary medicine, as these strains are often resistant to multiple classes of antimicrobials, severely limiting therapeutic options [5].

The global spread of MRSP has been well documented in both European and non-European countries, although prevalence rates and resistance patterns vary widely depending on geographical region, animal population, and local antimicrobial usage policies [6]. Despite growing awareness, significant gaps remain in our understanding of the epidemiology, molecular characteristics, and transmission dynamics of MRSP, as well as the identification of effective infection control and prevention strategies [7].

The emergence of MRSP parallels the broader worldwide expansion of multidrug-resistant bacterial pathogens. The first MRSP isolates were reported in Europe and North America in the early 2000s, and since then the organism has been detected across all continents [8,9]. Molecular epidemiological studies have identified the dissemination of specific epidemic clones, notably sequence type ST71 in Europe and ST68 in North America, which are often associated with extensive antimicrobial resistance and enhanced environmental persistence [10].

Several factors contribute to the successful spread of MRSP, including the widespread and sometimes inappropriate use of β-lactam antibiotics in companion animals, the presence of asymptomatic carriers—particularly dogs suffering from chronic dermatological conditions—and cross-transmission within veterinary clinics, grooming facilities, and households. These conditions facilitate both local and regional dissemination of resistant strains [11]. Although *S. pseudintermedius* is primarily regarded as an animal-associated pathogen, its zoonotic potential should not be overlooked [3,12,13]. An increasing number of human colonization and infection cases have been reported, particularly among individuals with close contact with companion animals, such as veterinarians, veterinary technicians, breeders, and pet owners [14,15]. Nevertheless, compared to *Staphylococcus aureus*, *S. pseudintermedius* appears to exhibit a lower degree of adaptation to the human host.

In this context, the present study aims to characterize the antimicrobial resistance profile of a *Staphylococcus pseudintermedius* strain isolated from a domestic dog affected by vulvovaginitis. By combining phenotypic and genomic approaches, this work seeks to contribute to a better understanding of MRSP infections and to support the development of improved diagnostic, therapeutic, and preventive strategies in line with the One Health concept.

## 2. Results

**Figure 1:**
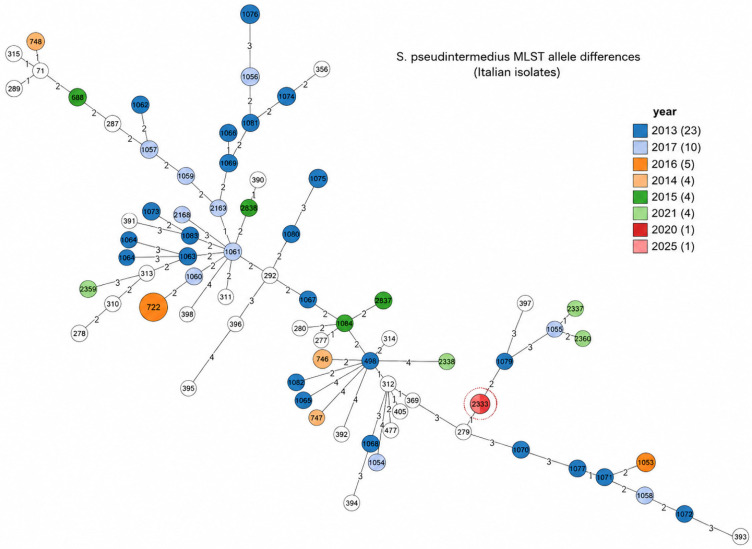
Minimum Spanning Tree for MLST allele differences among Italian *S. pseudintermedius* PubMLST genomes. Color nodes: sample collection year; grey number on branches: allele difference between node pair; integer number within node: MLST Sequence Type; red dashed circle: ST 2333 genomes, including the one described in this manuscript.

From aerobic bacterial cultures, after 24 h of incubation, shiny β-hemolytic colonies (2–3 mm diameter) with smooth surfaces and regular margins were observed. No growth of pathogenic microorganisms was observed in microaerophilic conditions. Further analysis by MALDI-TOF mass spectrometry of the isolated bacterial strains identified protein spectra referable to *S. pseudintermedius* with a score of 2.10. Species identification was also confirmed by in silico analysis, and subsequent MLST analysis assigned the isolate to* S. pseudintermedius* Sequence Type (ST) 2333 (Figure 1). Resistance pattern analysis identified an MRSP strain, which was susceptible only to amikacin, rifampicin, vancomycin and florfenicol, while being resistant to the antibiotics most commonly used in veterinary practice (Table 1). Whole-Genome sequencing (WGS) analysis confirmed the presence of multiple antimicrobial resistance (AMR) determinants. In particular, the *mecA* gene, responsible for methicillin resistance, was detected. Additional genes associated with resistance to other antimicrobial classes were identified, including determinants for β-lactams, aminoglycosides, macrolides–lincosamides-streptogramins (MLS), tetracyclines, trimethoprim and fluoroquinolones. In addition, a deep investigation of the *S. pseudintermedius* proteome provided evidence for two fluoroquinolone-resistance associated mutations within the DNA gyrase subunit A protein “*Gyr*A” and the DNA topoisomerase 4 subunit A “*Grl*A”, i.e., the S84L and S80I, respectively. The genomic resistome profile agreed with the multidrug-resistant (MDR) phenotype observed in vitro. The AMR genes predicted through bioinformatic analysis of the isolate’s genome are listed in Table 1.

## 3. Discussion

Methicillin resistance is mediated by the *mecA* gene, which encodes a penicillin-binding protein (PBP2a) with low affinity for β-lactams, conferring resistance to all penicillins, cephalosporins and carbapenems [16]. In addition, MRSP strains often exhibit a profile of multidrug resistance to other classes of antimicrobials, significantly limiting the available treatment options. This phenomenon complicates the clinical management of infections and raises important public health issues, considering the potential zoonotic transmission of the pathogen between domestic animals and humans [11]. In this context, we report a multidrug-resistant *S. pseudintermedius* with phenotypical resistance to antibiotics widely used in veterinary practice for small animals such as tetracyclines, macrolides, lincosamides, aminoglycosides, trimethoprim–sulfamethoxazole, fluoroquinolones and β-lactams.

The genotypic profile obtained by WGS strongly supports the phenotypic findings, highlighting the accumulation of resistance determinants within the same strain. The detection of multiple AMR genes suggests the presence of mobile genetic elements, such as SCCmec cassettes, which may facilitate horizontal gene transfer and spread in veterinary and domestic settings [17]. This aspect is particularly relevant considering the close contact between pets and humans.

In addition to the *mecA* gene, WGS revealed additional resistance genes that explain the multidrug-resistant phenotype. These included determinants associated with β-lactamase production (*blaZ*), macrolide–lincosamide resistance genes (*erm(B)*), tetracycline resistance genes (*tet(M)*, *tet(K)*), and trimethoprim resistance determinants (*dfrG*).

Our results are in line with the literature data, with the evidence that methicillin-resistant *Staphylococcus pseudintermedius* (MRSP) strains are resistant to β-lactam antibiotics through the expression of PBP2a, encoded by *mecA*, many isolates still retain and express the *blaZ–blaR1–blaI* locus. Indeed, it is widely accepted that staphylococci acquired the *blaZ–blaR1–blaI* gene cluster through mobile genetic elements as a consequence of selective pressure following the introduction of penicillin as an anti-staphylococcal therapy [18]. Furthermore, experimental evidence indicating that *blaI* contributes to staphylococcal resistance to host innate immune molecules and to virulence provides an additional explanation for why numerous methicillin-susceptible and methicillin-resistant *Staphylococcus pseudintermedius* isolates maintain the *blaZ–blaR1–blaI* genes [18].

The presence of the resistance genes *erm(B)*, *tet(M)*, *tet(K)*, and *dfrG* in *Staphylococcus pseudintermedius* highlights the growing prevalence of multidrug-resistant phenotypes, particularly in methicillin-resistant strains (MRSP). The *erm(B)* gene is responsible for methylation of the 23S rRNA, leading to cross-resistance to macrolides and lincosamides, which are antibiotics frequently used in veterinary practice. Similarly, resistance to tetracyclines is primarily mediated by *tet(M)*, which protects the ribosome from the drug’s action, while *tet(K)* contributes via an efflux mechanism.

Regarding trimethoprim, the *dfrG* gene represents one of the main resistance determinants, allowing the bacterium to circumvent the inhibition of folate synthesis [19].

Furthermore, the identification of the S84L substitution in *Gyr*A and the S80I substitution in *Grl*A highlights well-established mutations within the quinolone resistance-determining regions (QRDRs) of *Staphylococcus pseudintermedius* [20]. These amino acid changes are known to reduce fluoroquinolone binding affinity to DNA gyrase and topoisomerase IV, respectively, thereby impairing drug efficacy while preserving essential enzymatic functions [21]. This finding aligns with previous reports indicating that stepwise acquisition of QRDR mutations represents a key evolutionary pathway under antimicrobial selective pressure.

The co-occurrence of these genes in the same isolate suggests their possible association with mobile genetic elements, facilitating horizontal spread between different strains and species.

The accumulation of these genes within the same isolate reflects sustained selective pressure in the veterinary setting and contributes to therapeutic limitations.

These phenotypic and genotypic resistance profiles may explain the therapeutic failure with β-lactam antibiotics and, on the other hand, could lead veterinarians to use antimicrobials, such as amikacin and rifampicin, which are considered by the World Health Organisation to be “critically important antimicrobials” and should be reserved for the treatment of multidrug-resistant bacterial infections in human medicine [22].

This study also reports the first identification of *S. pseudintermedius* ST2333 in southern Italy [23]. Only one isolate with the same ST has been previously reported in the PubMLST database, isolated in northern Italy in 2020 (ID 2796) from a dog’s ear swab [24]. The assignment of the isolate to ST2333 demonstrates that it belongs to a genetically distinct lineage compared with the well-characterized epidemic MRSP clone ST71 circulating in Europe [25]. The identification of a relatively rare Sequence Type harboring an extensive resistome highlights the genetic heterogeneity of *S. pseudintermedius* and indicates that multidrug resistance is not limited to a small number of clones. This result highlights the high heterogeneity and dynamic population structure of *S. pseudintermedius*, in agreement with previous molecular epidemiology studies and contributing to a better understanding of the genetic diversity of this clinically relevant veterinary pathogen [26]. An additional aspect of interest concerns the One Health implications of these findings. While *S. pseudintermedius* is primarily an animal-associated species, sporadic human infections have been reported, indicating the potential for interspecies transmission [3,7,12,13,14,15,27]. The identification of MDR isolates in animals therefore emphasizes the importance of integrated surveillance approaches across veterinary and human health sectors. In fact, both the close proximity between owners and pets and the virulence characteristics similar to those of *S. aureus* could cause *S. pseudintermedius* infections to be more invasive and dangerous to human health. Therefore, therapeutic management should be based on accurate diagnosis, identification of underlying conditions, and assessment of severity. For mild or localized infections, for example, topical antiseptics should be preferred to reduce the use of systemic antibiotics, which should be reserved for clearly indicated cases. This approach could be important for limiting antimicrobial resistance. Indeed, the increasing prevalence of methicillin-resistant *S. pseudintermedius* and its capacity for interspecies gene transfer could further complicate infection control in both veterinary and human settings [28].

This study has some limitations that should be considered when interpreting the results. First, the investigation was based on a single clinical isolate, which limits the ability to draw broader epidemiological conclusions regarding the distribution of MRSP sequence type ST2333 in the regional canine population. Second, although whole-genome sequencing allowed the identification of antimicrobial resistance determinants, additional analyses—such as detailed characterization of mobile genetic elements, virulence factors, and SCCmec typing—were not performed and could provide further insights into the genetic background and transmission potential of the strain. Finally, no screening of animals or humans in close contact with the affected dog was conducted; therefore, the possible circulation or transmission of this lineage within the household or veterinary environment could not be evaluated. Future studies including larger numbers of isolates and integrated epidemiological investigations will be necessary to better understand the distribution and clinical relevance of this MRSP lineage.

## 4. Materials and Methods

### 4.1. Case Description

A vaginal swab from an 11-year-old English Setter with vulvovaginitis, without discharge, and a history of unsuccessful antibiotic treatment was submitted to the diagnostic laboratories of the Istituto Zooprofilattico Sperimentale of Puglia and Basilicata. The swab was seeded on Columbia agar plates and incubated at 37 °C under aerobic and microaerophilic conditions (5% CO_2_).

### 4.2. Sample Preparation for MALDI-TOF MS Analysis

The identification of all strains grown on media was carried out as previously described [29]. Briefly, isolates were picked with a toothpick and spread onto a 96-well steel plate. Afterwards, 1 μL of α-cyano-4-hydroxycinnamic acid was added to each sample (HCCA, Bruker Daltonik GmbH, Bremen, Germany). The plate thus prepared was appropriately analyzed using a Microflex LT/SH™ mass spectrometer (Bruker Daltonics GmbH & Co KG., Bremen, Germany). The data were automatically processed by the MBT Compass 4.1.70 software (Bruker Daltonik GmbH, Bremen, Germany) and the mass spectra were compared with those of known microbial isolates from the commercial libraries provided by Bruker Daltonik (MBT Compass library v 7.0. 0.0).

### 4.3. Antimicrobial Susceptibility Testing

The strain of *Staphylococcus pseudintermedius* was characterised phenotypically via broth microdilution using precasted plates for Gram + pathogens (Thermo Fisher Diagnostics, Paisley, UK). The antibiotics tested were amikacin (1–64 µg/mL), chloramphenicol (2–16 µg/mL), clindamycin (0.12–8 µg/mL), doxycycline (0.12–16 µg/mL), enrofloxacin (0.06–4 µg/mL), erythromycin 0.12–8 µg/mL, florfenicol (4–8 µg/mL), gentamicin (2–16 µg/mL), kanamycin (8–16 µg/mL), oxacillin + 2% NaCl (0.25–8 µg/mL), penicillin (0.06–16 µg/mL), rifampicin (0.5–4 µg/mL), trimethoprim/sulfamethoxazole (0.25/4.75–4/76 µg/mL), tetracycline (0.12–16 µg/mL) and vancomycin (0.25–32 µg/mL). The quality control of the batch was performed with *Staphylococcus aureus* ATCC 29213. The antimicrobial test was carried out according to the manufacturer’s instructions. The definition of sensitivity or resistance was based on the CLSI VET01S 7th edition breakpoints [30].

### 4.4. Whole-Genome Sequencing and Typing

Genomic DNA was extracted from a single bacterial colony of *S. pseudintermedius* using the QIAmp DNA mini kit (Qiagen, Hilden, Germany), according to the manufacturer’s instructions. DNA concentration was measured using a Qubit™ 3.0 Fluorometer with the Qubit™ dsDNA HS Assay Kit (Thermo Fisher Scientific, Waltham, MA, USA). Paired-end sequencing libraries were prepared with the Illumina DNA Prep Kit (Illumina, San Diego, CA, USA), and whole-genome sequencing was carried out on an Illumina MiSeq platform (2 × 250 bp) [31]. Raw sequencing reads were subjected to quality control and adapter trimming using fastp v1.1.0. [32]. De novo genome assembly was performed with Shovill v1.4.2. [33]. Assembly quality metrics and potential contamination were evaluated using QUAST v5.3.0 [34] and CheckM2 (taxonomy_wf) v1.2.4 [35], respectively. Species identification was performed by using GTDB-Tk v.2.5.2 [36] (db release: 220). Genotyping for the assembled genome was subsequently performed by conventional Multi-Locus Sequence Typing (MLST) scheme via the PubMLST website [24,37,38]. The resulting MLST allele profile was compared with public data, by using GrapeTree PubMLST plugin [39]. Genes associated with antimicrobial resistance were first identified using AMRFinder2 [40]. In addition, BAKTA [41] was used to predict the whole proteome, while possible antimicrobial-resistance associated amino acid substitutions were searched through BLASTP [42] on ClusteredNR NCBI database.

## 5. Conclusions

In conclusion, this study describes the phenotypic and genomic characterization of a multidrug-resistant MRSP ST2333 isolate recovered from a canine vulvovaginitis case in southern Italy. The accumulation of multiple antimicrobial resistance determinants within a genetically distinct lineage highlights the ongoing diversification of MRSP populations. The integration of antimicrobial susceptibility testing with whole-genome sequencing provides a robust framework for surveillance and supports antimicrobial stewardship strategies in veterinary medicine. Strengthening coordinated monitoring efforts within a One Health approach will be critical to limit the dissemination of multidrug-resistant staphylococci.

## Figures and Tables

**Table 1:** Resistance to single antimicrobial agents used in veterinary medicine for *S. pseudintermedius* strain. S: Sensitive; I: Intermediate; R: Resistant; MIC: Minimum inhibitory concentration.

Antimicrobial Agents		Breakpoints (µg/mL)				Results	Associated Predicted Genes
		S≤	I	R≥	MIC		
AMI	Amikacin	4	8	16	2	S	
CHL	Chloramphenicol	2	4	8	>16	**R**	*catA*
CLI	Clindamycin	0.5	1–2	4	>2	**R**	*erm(B)*
DOX	Doxycycline	0.12	0.25	8	8	**R**	*tet(M)*, *tet(K)*
ENRO	Enrofloxacin	0.5	1–2	4	>4	**R**	*gyrA*, *grlA*
ERY	Erythromycin	0.5	1–4	8	>8	**R**	*erm(B)*
FFN	Florfenicol	8		16	≤4	S	
GEN	Gentamicin	4	8	16	8	**I**	*aac(6′)-Ie/aph(2″)-Ia*
KAN	Kanamicin	8	--	16	>16	**R**	*aph(3′)-IIIa*
OXA+	Oxacillin+ 2% NaCl	0.5	--	1	>4	**R**	*mecA*, *blaI, blaR1*, *blaZ*
PEN	Penicillin	0.12	--	0.25	>16	**R**	*mecA*, *blaI*, *blaR1*, *blaZ*
RIF	Rifampicin	1	2	4	≤0.5	S	
SXT	Trimethoprim/Sulfamethoxazole	2/38	--	4/76	>4/76	**R**	*dfrG*
TET	Tetracycline	0.25	0.5	1	>16	**R**	*tet(M)*, *tet(K)*
VAN	Vancomycin	4	8–16	32	1	S	

## Data Availability

All data generated or analyzed during this study are included in this published article: genome sequence has been submitted on NCBI “https://www.ncbi.nlm.nih.gov/bioproject/PRJNA1445790 (accessed on 1 April 2026)” and PubMLST “https://pubmlst.org/bigsdb?db=pubmlst_spseudintermedius_isolates; id: 3485 (accessed on 19 February 2026)”.

## References

[1] Kavya Sudha A. (2025). A Review on *Staphylococcus pseudintermedius*. Int. J. Sci. Res. Manag..

[2] Bannoehr J., Guardabassi L. (2012). *Staphylococcus pseudintermedius* in the dog: Taxonomy, diagnostics, ecology, epidemiology and pathogenicity. Vet. Dermatol..

[3] Moses I.B., Santos F.F., Gales A.C. (2023). Human Colonization and Infection by *Staphylococcus pseudintermedius*: An Emerging and Underestimated Zoonotic Pathogen. Microorganisms.

[4] Hillier A., Lloyd D.H., Weese J.S., Blondeau J.M., Boothe D., Breitschwerdt E., Guardabassi L., Papich M.G., Rankin S., Turnidge J.D. (2014). Guidelines for the diagnosis and antimicrobial therapy of canine superficial bacterial folliculitis (Antimicrobial Guidelines Working Group of the International Society for Companion Animal Infectious Diseases). Vet. Dermatol..

[5] Breyer G.M., Saggin B.F., de Carli S., da Silva M.E.R.J., da Costa M.M., Brenig B., Azevedo V.A.d.C., Cardoso M.R.d.I., Siqueira F.M. (2023). Virulent potential of methicillin-resistant and methicillin-susceptible *Staphylococcus pseudintermedius* in dogs. Acta Trop..

[6] Pires Dos Santos T., Damborg P., Moodley A., Guardabassi L. (2016). Systematic Review on Global Epidemiology of Methicillin-Resistant *Staphylococcus pseudintermedius*: Inference of Population Structure from Multilocus Sequence Typing Data. Front. Microbiol..

[7] Besharati R., Haghbin A., Hashemi S.A., Vosoughi-Motlagh A., Azimian A. (2024). Molecular detection and characterization of methicillin-resistant Staphylococcus pseudintermedius (MRSP) ST2361 in a healthcare setting. One Health.

[8] Menandro M.L., Dotto G., Mondin A., Martini M., Ceglie L., Pasotto D. (2019). Prevalence and characterization of methicillin-resistant *Staphylococcus pseudintermedius* from symptomatic companion animals in Northern Italy: Clonal diversity and novel sequence types. Comp. Immunol. Microbiol. Infect. Dis..

[9] Nocera F.P., Parisi A., Corrente M., De Martino L. (2020). Evidence of new sequence types of methicillin-resistant *Staphylococcus pseudintermedius* in Italy. Pol. J. Vet. Sci..

[10] Grist L.F., Brown A., Fitzpatrick N., Mariano G., La Ragione R.M., Van Vliet A.H.M., Mehat J.W. (2025). Global phylogenomic analysis of *Staphylococcus pseudintermedius* reveals genomic and prophage diversity in multidrug-resistant lineages. Microb. Genom..

[11] Nocera F.P., De Martino L. (2024). Methicillin-resistant *Staphylococcus pseudintermedius*: Epidemiological changes, antibiotic resistance, and alternative therapeutic strategies. Vet. Res. Commun..

[12] Bhooshan S., Negi V., Khatri P.K. (2020). *Staphylococcus pseudintermedius*: An undocumented, emerging pathogen in humans. GMS Hyg. Infect. Control.

[13] Roberts E., Nuttall T.J., Gkekas G., Mellanby R.J., Fitzgerald E., Paterson G.K. (2024). Not just in man’s best friend: A review of *Staphylococcus pseudintermedius* host range and human zoonosis. Res. Vet. Sci..

[14] Asleh M., Feinstein Y., Lazar I., Rokney A., Baum M., Sagi O., Leibovitz E., Danino D. (2022). Severe Pneumonia Caused by Methicillin-Resistant *Staphylococcus pseudintermedius* in an Oncology Patient: Case Report and Literature. Rev. Microb. Drug Resist..

[15] Somayaji R., Priyantha M.A., Rubin J.E., Church D. (2016). Human infections due to *Staphylococcus pseudintermedius*, an emerging zoonosis of canine origin: Report of 24 cases. Diagn. Microbiol. Infect. Dis..

[16] MacFadyen A.C., Paterson G.K. (2024). Methicillin resistance in *Staphylococcus pseudintermedius* encoded within novel staphylococcal cassette chromosome mec (SCCmec) variants. J. Antimicrob. Chemother..

[17] Oliveira D.C., de Lencastre H. (2002). Multiplex PCR strategy for rapid identification of structural types and variants of the mec element in methicillin-resistant *Staphylococcus aureus*. Antimicrob. Agents Chemother..

[18] Pence M.A., Haste N.M., Meharena H.S., Olson J., Gallo R.L., Nizet V., Kristian S.A. (2015). Beta-Lactamase Repressor BlaI Modulates *Staphylococcus aureus* Cathelicidin Antimicrobial Peptide Resistance and Virulence. PLoS ONE.

[19] Elbehiry A., Marzouk E. (2025). Staphylococci in Livestock: Molecular Epidemiology, Antimicrobial Resistance, and Translational Strategies for One Health Protection. Vet. Sci..

[20] Ostrer L., Khodursky R.F., Johnson J.R., Hiasa H., Khodursky A. (2019). Analysis of mutational patterns in quinolone resistance-determining regions of GyrA and ParC of clinical isolates. Int. J. Antimicrob. Agents.

[21] Myrenås M., Pedersen K., Windahl U. (2024). Genomic Analyses of Methicillin-Resistant *Staphylococcus pseudintermedius* from Companion Animals Reveal Changing Clonal Populations, Multidrug Resistance, and Virulence. Antibiotics.

[22] WHO List of Medically Important Antimicrobials. https://cdn.who.int/media/docs/default-source/gcp/who-mia-list-2024-lv.pdf?sfvrsn=3320dd3d_2.

[23] Ventrella G., Moodley A., Grandolfo E., Parisi A., Corrente M., Buonavoglia D., Guardabassi L. (2017). Frequency, antimicrobial susceptibility and clonal distribution of methicillin-resistant *Staphylococcus pseudintermedius* in canine clinical samples submitted to a veterinary diagnostic laboratory in Italy: A 3-year retrospective investigation. Vet. Microbiol..

[24] *Staphylococcus pseudintermedius* Isolates Database. https://pubmlst.org/organisms/staphylococcus-pseudintermedius.

[25] Giarratana L.R., Pirolo M., Roch F.F., Conrady B., Guardabassi L. (2026). Evolving landscape of methicillin-resistant *Staphylococcus pseudintermedius*: The emergence of new epidemic waves across Europe, Asia and North America. J. Antimicrob. Chemother..

[26] Sawhney S.S., Vargas R.C., Wallace M.A., Muenks C.E., Lubbers B.V., Fritz S.A., Burnham C.D., Dantas G. (2023). Diagnostic and commensal *Staphylococcus pseudintermedius* genomes reveal niche adaptation through parallel selection of defense mechanisms. Nat. Commun..

[27] Youn J.H., Moodley A., Park Y.H., Sugimoto C. (2013). Genome Sequence of Methicillin-Resistant *Staphylococcus pseudintermedius* Sequence Type 233 (ST233) Strain K7, of Human Origin. Genome Announc..

[28] Bellato A., Robino P., Stella M.C., Scarrone L., Scalas D., Nebbia P. (2022). Resistance to Critical Important Antibacterials in *Staphylococcus pseudintermedius* Strains of Veterinary Origin. Antibiotics.

[29] Rondinone V., Palazzo L., Bianco A., La Rosa G., Manzulli V., Galante D., Del Sambro L., Toce M., Romano A.C., Pace L. (2025). Identification of *Actinobacillus seminis* as the cause of abortion in sheep by matrix-assisted laser desorption/ionization time-of-flight mass spectrometry and whole genome sequencing. Ital. J. Food Saf..

[30] Fraccalvieri R., Bianco A., Difato L.M., Capozzi L., Del Sambro L., Castellana S., Donatiello A., Serrecchia L., Pace L., Farina D. (2025). Isolation and Characterization of Colistin-Resistant *Enterobacteriaceae* from Foods in Two Italian Regions in the South of Italy. Microorganisms.

[31] Bianco A., Capozzi L., Monno M.R., Del Sambro L., Manzulli V., Pesole G., Loconsole D., Parisi A. (2021). Characterization of *Bacillus cereus* Group Isolates From Human Bacteremia by Whole-Genome Sequencing. Front. Microbiol..

[32] Chen S., Zhou Y., Chen Y., Gu J. (2018). fastp: An ultra-fast all-in-one FASTQ preprocessor. Bioinformatics.

[33] Seemann T. (2016). Shovill Github. https://github.com/tseemann/shovill.

[34] Gurevich A., Saveliev V., Vyahhi N., Tesler G. (2013). QUAST: Quality assessment tool for genome assemblies. Bioinformatics.

[35] Chklovski A., Parks D.H., Woodcroft B.J., Tyson G.W. (2023). CheckM2: A rapid, scalable and accurate tool for assessing microbial genome quality using machine learning. Nat. Methods.

[36] Chaumeil P.A., Mussig A.J., Hugenholtz P., Parks D.H. (2019). GTDB-Tk: A toolkit to classify genomes with the Genome Taxonomy Database. Bioinformatics.

[37] Jolley K.A., Bray J.E., Maiden M.C.J. (2018). Open-access bacterial population genomics: BIGSdb software, the PubMLST.org website and their applications. Wellcome Open Res..

[38] Solyman S.M., Black C.C., Duim B., Perreten V., van Duijkeren E., Wagenaar J.A., Eberlein L.C., Sadeghi L.N., Videla R., Bemis D.A. (2013). Multilocus sequence typing for characterization of *Staphylococcus pseudintermedius*. J. Clin. Microbiol..

[39] Zhou Z., Alikhan N.F., Sergeant M.J., Luhmann N., Vaz C., Francisco A.P., Carriço J.A., Achtman M. (2018). GrapeTree: Visualization of core genomic relationships among 100,000 bacterial pathogens. Genome Res..

[40] Feldgarden M., Brover V., Haft D.H., Prasad A.B., Slotta D.J., Tolstoy I., Tyson G.H., Zhao S., Hsu C.-H., McDermott P.F. (2019). Validating the AMRFinder Tool and Resistance Gene Database by Using Antimicrobial Resistance Genotype-Phenotype Correlations in a Collection of Isolates. Antimicrob. Agents Chemother..

[41] Schwengers O., Jelonek L., Dieckmann M.A., Beyvers S., Blom J., Goesmann A. (2021). Bakta: Rapid and standardized annotation of bacterial genomes via alignment-free sequence identification. Microb. Genom..

[42] Altschul S.F., Gish W., Miller W., Myers E.W., Lipman D.J. (1990). Basic local alignment search tool. J. Mol. Biol..

